# Persistent *Wolbachia* and Cultivable Bacteria Infection in the Reproductive and Somatic Tissues of the Mosquito Vector *Aedes albopictus*

**DOI:** 10.1371/journal.pone.0006388

**Published:** 2009-07-27

**Authors:** Karima Zouache, Denis Voronin, Van Tran-Van, Laurence Mousson, Anna-Bella Failloux, Patrick Mavingui

**Affiliations:** 1 Université de Lyon, Lyon, France; 2 Université Lyon 1, Villeurbanne, CNRS, UMR5557, Ecologie Microbienne, Lyon, France; 3 Institut Pasteur, Génétique moléculaire des Bunyavirus, Paris, France; University of Hyderabad, India

## Abstract

**Background:**

Commensal and symbiotic microbes have a considerable impact on the behavior of many arthropod hosts, including hematophagous species that transmit pathogens causing infectious diseases to human and animals. Little is known about the bacteria associated with mosquitoes other than the vectorized pathogens. This study investigated *Wolbachia* and cultivable bacteria that persist through generations in *Ae. albopictus* organs known to host transmitted arboviruses, such as dengue and chikungunya.

**Methodology/Principal Findings:**

We used culturing, diagnostic and quantitative PCR, as well as *in situ* hybridization, to detect and locate bacteria in whole individual mosquitoes and in dissected tissues. *Wolbachia*, cultivable bacteria of the genera *Acinetobacter*, *Comamonas*, *Delftia* and *Pseudomonas* co-occurred and persisted in the bodies of both males and females of *Ae. albopictus* initially collected in La Réunion during the chikungunya outbreak, and maintained as colonies in insectaries. In dissected tissues, *Wolbachia* and the cultivable *Acinetobacter* can be detected in the salivary glands. The other bacteria are commonly found in the gut. Quantitative PCR estimates suggest that *Wolbachia* densities are highest in ovaries, lower than those of *Acinetobacter* in the gut, and approximately equal to those of *Acinetobacter* in the salivary glands. Hybridization using specific fluorescent probes successfully localized *Wolbachia* in all germ cells, including the oocytes, and in the salivary glands, whereas the *Acinetobacter* hybridizing signal was mostly located in the foregut and in the anterior midgut.

**Conclusions/Significance:**

Our results show that *Proteobacteria* are distributed in the somatic and reproductive tissues of mosquito where transmissible pathogens reside and replicate. This location may portend the coexistence of symbionts and pathogens, and thus the possibility that competition or cooperation phenomena may occur in the mosquito vector *Ae. albopictus*. Improved understanding of the vectorial system, including the role of bacteria in the vector's biology and competence, could have major implications for understanding viral emergences and for disease control.

## Introduction

Mosquitoes are medically important arthropod vectors of vertebrate pathogens. For instance, *Aedes albopictus*, and its sister taxon *Aedes aegypti*, are vectors of a large number of arboviruses, notably dengue and chikungunya [1]. Since 2005, La Réunion and neighboring islands in the Indian Ocean have experienced severe epidemics of chikungunya involving high incidences in the population [http://www.invs.sante.[fr, 2]]. The isolation and sequencing of the chikungunya virus from patients in La Réunion during a massive disease outbreak have revealed a prevalence of clinical isolates harboring nucleotide changes in both structural and non-structural loci; one particular mutation was found in glycoprotein E1, in a region predicted to interact with the target membrane [3]. Entomological field surveys [4], [5] and vector competence assays in the laboratory [6] have demonstrated that *Ae. albopictus*, which is much more anthropophilic than *Ae. aegypti* in La Réunion, is a very efficient vector.

In the absence of effective vaccines, arbovirus transmission can only be reduced by limiting mosquito densities by the mechanical reduction of breeding sites and by the application of insecticides. Unfortunately, insecticides impact non-target insects as well, and most mosquito species have developed resistance [7]. There is increasing interest in the use of microbes associated with arthropod vectors to interfere with the transmission of pathogens with a view to overcoming these difficulties by sustainable approaches [8]. Indeed, if on the one hand, microbial symbionts can confer a fitness gain on their arthropod hosts, including better nutrition [9], heat tolerance [10], [11], and resistance to pathogens [12], [13], [14], on the other hand, arthropod microbiota can be pathogenic for the host vector [15], [16], or can have deleterious effects on host reproduction [17], [18]. Finally, microbes associated with arthropods can either enhance or weaken vector competence [19], [20], [21]. Consequently, interference with one or more of these aspects of host behavior by natural or transgenic microbes could be exploited to manage arthropod vector-borne diseases by an approach known as “paratransgenesis” [22], [23].

Despite the importance of microbes in the ecology and behavior of many arthropods [24], including hematophagous vectors such as ticks [25], tsetse flies [26] and lice [27], little is known about the mosquito-associated microbiota. Most of the few studies that have investigated the bacterial communities of *Culex* and *Anopheles* mosquitoes have focused on the midgut compartment [28], [29], [30], [31], 32, 34, 35, 36. Very little is known about *Aedes*-associated bacteria. DeMaio and co-workers [37] were the first to report the midgut bacterial flora of wild *Aedes triseriatus*. Recently, members of the *Bacillus* and *Serratia* genera have been identified in the larval gut [38], and adult ventral diverticulum [39] of *Ae. aegypti*, respectively. Attempts have been made to use the gut-inhabiting bacteria to interfere with parasite transmission in mosquitoes [40], [41], [42]. In *Ae. albopictus*, the obligate intracellular bacterium *Wolbachia* has mainly been looked for in laboratory colonies and field-caught individuals [43], [44]. This bacterium induces cytoplasmic incompatibility [45], [46], [47], [48] that causes embryogenic death, a feature that could be exploited to control insect pests [17]. More studies are needed to make a complete inventory of the microbial communities of *Ae. albopictus*, and identify taxa that could be manipulated for paratransgenesis purposes. In this study, we investigated the presence and location of *Wolbachia* and of cultivable bacteria in a colony of *Ae. albopictus*, collected during the explosive chikungunya epidemics in La Réunion, and maintained under laboratory conditions since 2006. Culturing and PCR-based techniques coupled with *in situ* hybridization were used to detect *Wolbachia* and cultivable bacterial genera differentially distributed in somatic and reproductive tissues.

## Results

### Characteristics of the dominant *Proteobacteria*

A total of 3 to 100 CFU were found per early emerging *Ae. albopictus* mosquito. Eight colony types were obtained in the two media used; notably two types from male mosquitoes and six types from females. Two representatives of each colony type were used for genomic DNA extraction and PCR amplification of the *rrs* gene using universal primers (Table 1). Amplified rDNA restriction analysis (ARDRA) of the amplified *rrs* genes revealed five distinct patterns (not shown), the corresponding PCR products of which were fully sequenced. Blastn analysis (Table 2) identified two nearly complete *rrs* gene sequences as being closely related to uncultured *Comamonas* spp. (99% similarity), and the other three were affiliated to three species, *Acinetobacter calcoaceticus* (99% similarity), *Delftia* sp. (99% similarity), and *Pseudomonas alcaligenes* (99% similarity). Isolates of the genera *Comamonas*, *Delftia* and *Pseudomonas* were recovered from females, whereas *Acinetobacter* isolates were obtained from males.

**Table 1:** Primers used in this study.

Group	Gene	Primer name	Primer sequence (5′–3′)	Amplicon size/Tm	References
**Organism**					
*Eubacteria*	*rrs*	pA	5′ AGAGTTTGATCCTGGCTCAG 3′	1500/55	[83]
		pH	5′ AAGGAGGTGATCCAGCCGCA 3′		
	*rrs*	16S (V3) 338F	5′ GCCGCCCGCCGCGCGCGGCGGGCGGGGCGGGGGCACGGGGGGACTCCTACGGGAGGCAGCAG 3′	variable	[84]
		16S (V3) 520R	5′ATTACCGCGGCTGCTGG 3′		
*Wolbachia*	*rrs*	99F	5′ TTGTAGCCTGCTATGGTATAACT 3′	864/52	[85]
		1994R	5′ GAATAGGTATGATTTTCATGT 3′		
	*wsp*	81F	5′ TGGTCCAATAAGTGTATGAAGAAAC 3′	600/55	[86]
		183F	5′ AAGGAACCGAAGTTCATG 3′	508/52	[87]
		328F	5′ CCAGCAGATACTATTGCG 3′	363/52	[87]
		691R	5′ AAAGGGGACTGATGATGT 3′		[87]
					
*Comamonas*	*rrs*	Com199F	5′ CCTTGTGCTACTAGAGC 3′	433/53	This study
		Com614R	5′ GCAGTCACAATGGCAGTT 3′		This study
*Delftia*	*rrs*	Delf63F	5′ TAACAGGTCTTCGGACGC 3′	397/56	This study
		Delf440R	5′ CCCCTGTATTAGAAGAAGCT 3′		This study
*Pseudomonas*	*rrs*	Ps For	5′ GGTCTGAGAGGATGATCAGT 3′	990/52	[88]
		Ps Rev	5′ TTAGCTCCACCTCGCGGC 3′		
*Acinetobacter*	*rrs*	Acine1	5′ ACTTTAAGCGAGGAGGAGGCT 3′	426/58	[80]
		Ac	5′ GCGCCACTAAAGCCTCAAAGGCC 3′		[82]
**Plasmid**					
pQuantAlb	*wsp* wAlbA	QADir1	5′ GGGTTGATGTTGAAGGAG 3′	264/60	[77]
		QArev2	5′ CACCAGCTTTTACTTGACC 3′		[77]
	*wsp* wAlbB	183F	5′ AAGGAACCGAAGTTCATG 3′	112/60	[87]
		QBrev2	5′ AGTTGTGAGTAAAGTCCC 3′		[77]
	*actin*	ActAlb-dir	5′ GCAAACGTGGTATCCTGAC 3′	139/60	[77]
		ActAlb-rev	5′ GTCAGGAGAACTGGGTGCT 3′		
TOPO 2.1	*rrs Acinetobacter*	ACA	5′ TAGAGTGTGGGAGAGGAT 3′	208/60	[81]
		Ac	5′ GCGCCACTAAAGCCTCAAAGGCC 3′		[82]

**Table 2:** Bacterial community of *Aedes albopictus*.

Sample type	Name of clone/Band number	Size (bp)	Accession number	Phylogenetic affiliation	Closest relative organism	Accession number	No identical/total similarity (%)
**Cultivable bacteria**							
	KZ-OAlF1^a^	1525	FJ688377	*βetaproteobacteria*	Uncultured *Comamonas* sp. clone DS104	DQ234187.2	1524/1525 (99)
	KZ-OAlF2^a^	1525	FJ688376	*βetaproteobacteria*	*Delftia* sp. 332	EU888308.1	1524/1525 (99)
	KZ-OAlF3^a^	1529	FJ688378	*Gammaproteobacteria*	*Pseudomonas alcaligenes* strain S3	DQ115541.1	1490/1495 (99)
	KZ-OAlM b	1529	FJ688379	*Gammaproteobacteria*	*Acinetobacter calcoaceticus* type strain NCCB 22016	AJ888983.1	1513/1515 (99)
**DDGE**							
	[1; 2; 14; 15]a, b, c, d, e	169	GQ290053	*Alphaproteobacteria*	*Wolbachia* sp. wRi, complete genome	CP001391.1	169/169 (100)
	[3; 4; 16]a, b, d, e	194	FJ688377	*βetaproteobacteria*	Uncultured *Comamonas* sp. clone DS104	FJ950572.1	194/194 (100)
	[5. 13] a, b, d, e	194	GQ290055	*Gammaproteobacteria*	*Pseudomonas stutzeri* strain Bon_b1	FN397902.1	194/194 (100)
	[6; 7] a, b, d, e	194	GQ290057	*Gammaproteobacteria*	*Strenotrophomonas maltophilia* strain d402	FJ950659.1	194/194 (100)
	8 a, b, d	194	FJ688376	*βetaproteobacteria*	*Delftia* sp. 332	EU888308.1	194/194 (100)
	9 c, d	169	GQ290056	*Alphaproteobacteria*	*Mesorhizobium loti* strain U261	DQ310706.1	166/169 (98)
	10 a, b, d	195	FJ688379	*Gammaproteobacteria*	*Acinetobacter calcoaceticus* type strain NCCB 22016	AJ888983.1	195/195 (100)
	[11; 12] d	194	GQ290058	Unknown	Uncultured bacterium clone 16saw44-1d03.p1k	EF6044192.1	194/194 (100)

a Female individuals.

b male individuals.

c ovaries.

d gut.

e salivary glands.

To obtain an overview of the total bacterial community, PCR-DGGE fingerprints of samples from whole insects and from dissected tissues were produced using specific *rrs* -gene primers and the corresponding hypervariable V3 regions (Figure S1). Bands were gel-excised and re-amplified. Direct sequencing of the PCR product generated in some cases double sequences, indicating the presence of more than one V3 in a particular excised band. These were excluded from the analysis. Among the single sequences, blast analysis identified an uncultivable bacterium, as well as the genera *Mesorhizobium* and *Stenotrophomonas* (Table 2). The presence of sequences affiliated with *Wolbachia* and with the four cultivable genera (*Acinetobacter, Comamonas, Delftia* and *Pseudomonas*) was also found.

Amplification with specific primers was performed to further explore *Wolbachia* and the dominant cultivable bacteria in the insect tissues. Positive PCR signals corresponding to *Wolbachia* strains *w*AlbA and *w*AlbB were obtained for all three of the organs tested (salivary glands, ovaries, and gut), as well as in the eggs, for four generations (Table 3), confirming the “invasive behavior” of this vertically-transmitted bacterial genus. The genus *Acinetobacter* was detected in the gut and salivary glands, whereas PCR products corresponding to *Comamonas*, *Delftia* and *Pseudomonas* were obtained only in the gut. Sequencing the amplified fragments confirmed the identity of each targeted bacterium (not shown). In the subsequent experiments, we focused on *Wolbachia* and *Acinetobacter*, which were detected in both gut and salivary glands.

**Table 3:** Genus-specific PCR^a^ amplifications in whole body and organs^b^.

	Whole body male and female	Gut	Salivary glands	Ovaries	Eggs
*Eubacteria*	+	+	+	+	+
*Wolbachia*	+	+	+	+	+
*Acinetobacter*	+	+	+	−	−
**Pseudomonas**	+	+	−	−	−
*Comamonas*	+	+	−	−	−
*Delftia*	+	+	−	−	−

a Primers used are listed in Table 1. Identity of products was confirmed by sequencing.

b Pools of 10 organs from females were tested in three biological replicates.

#### Densities of *Wolbachia* and *Acinetobacter* in mosquito organs

The numbers of the bacterial cells varied depending on the genus and on the targeted organs (Table 4). The relative density (number of *wsp* gene per host *actin* gene) of *Wolbachia* was higher (*P*<0.005) in ovaries than in the gut and salivary glands. No differences (*P*>0.05) were found between the two *Wolbachia w*AlbA and *w*AlbB strains in all the three organs. The highest density of *Acinetobacter* was found in the gut (*P*<0.001), outnumbering *Wolbachia* as well *(P*<0.005). Densities of *Wolbachia* and *Acinetobacter* were not significantly different in the salivary glands (*P*>0.05).

**Table 4:** Bacterial density in female *Ae. albopictus* organs.

Organs	No. of *Wolbachia* (10^2^) per 10 organs		No. of *Wolbachia wsp* gene/No. of Ae. albopictus actin gene		No. of *Acinetobacter* (10^2^) per 10 organs	No. of *Acinetobacter rrs*/No. *Ae. albopictus actin* gene
	*w*AlbA	*w*AlbB	*w*AlbA	*w*AlbB		
Ovary	27.54±1.31^a^	28.80±2.21^a^	2.62±0.13^A^	2.71±0.21^A^	nd	nd
SG	0.19±0,02^b^	0.089±0.007^b^	0.035±0.004^B^	0.017±0.0015^B^	1.58±0.44^c^	0.03±0.001^B^
Gut	2.83±0.16^c^	2.28±0.2^c^	0.15±0.0087^C^	0.13±0.01^C^	2.67±0.35^c^	0.33±0,04^D^

Statistical analysis was performed on log-transformed values. The dependent *t*-test was used to compare two means. Since multiple and non-independent tests were performed, the exact risk of rejecting a true null hypothesis is hard. For safety, we chose to reject H0 at *P*<0.005. Mean values±SE marked with the same letter are not significantly different (*P*>0.005).

#### Localization of bacteria in dissected tissues

**Figure 1:**
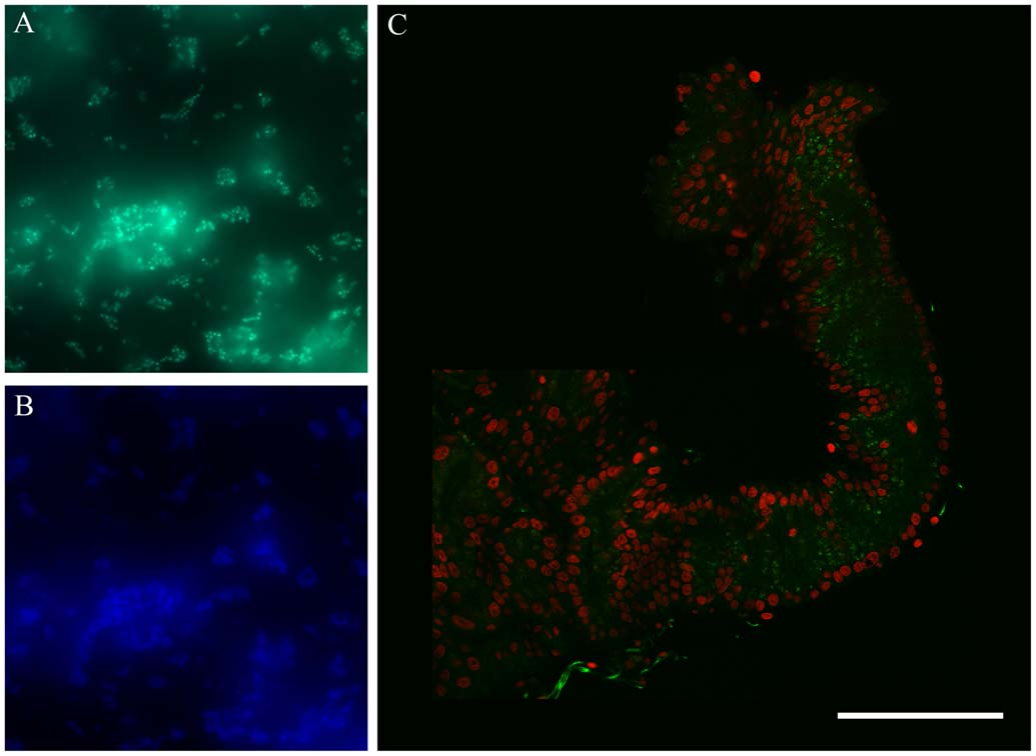
Microscopic views of *Acinetobacter* and infected mosquito tissues. FISH with a specific oligonucleotide probe (A) and DAPI (B) targeting *Acinetobacter calcoaceticus* grown in a pure culture. (C) *Aedes albopictus* gut infected with *Acinetobacter calcoaceticus* (green). Nuclei are stained with propidium iodide (red). A and B, magnification 100X; C, bar 500 µm.

**Figure 2:**
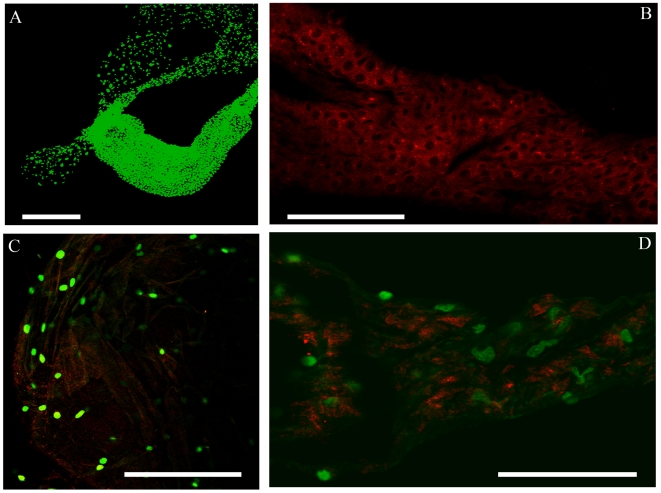
Confocal microscopy of *Aedes albopictus* salivary glands infected with *Wolbachia*. (A) General view of salivary glands (SG) showing cell nuclei stained by SYTOX (green). *Wolbachia* (red dots) are detected by the *rrs* specific probe in the cells of the median canal (B) and lateral lobes (C, D). Nuclei are in green. Bar, 500 µm.

To localize the bacteria in mosquito tissues, FISH genus-specific probes available for *Acinetobacter* and *Wolbachia* were used. To do this, FISH probes were first tested using *Acinetobacter calcoaceticus* isolate KZ-OAlM cultured in rich medium, and *Wolbachia* strain *w*AlbB hosted in *Ae. albopictus* cell line Aa23. Specific signals were detected for both *Acinetobacter* (Fig. 1A) and *Wolbachia* (not shown). The probes were then hybridized against the dissected tissues using three independent biological samples. Confocal microscopic observations of somatic tissues showed hybridizing signals for *Acinetobacter* in the inner surface of epithelial cells and in lumen space of the foregut and the anterior midgut (Fig. 1C). These signals were observed in all 10 of the dissected guts of females from generations F2 to F5. *Acinetobacter* could not be detected in the cell cytoplasm or basal or ventral parts of the epithelial cells, suggesting that this bacterial genus is mainly located in the intervillous space. No significant *Acinetobacter* signal was detected in the central part of midgut or hindgut. *Wolbachia* probes detected signals in the cytoplasm of salivary gland cells (Fig. 2). The medium lobe displayed relatively low signal intensity (Fig. 1B) compared to high hybridizing dots found in the lateral lobes (Fig. 2C and D). In contrast to the positive PCR results (see above), no significant fluorescent signals were observed in the gut for *Wolbachia*, nor in the ovary for *Acinetobacter* (not shown).

**Figure 3:**
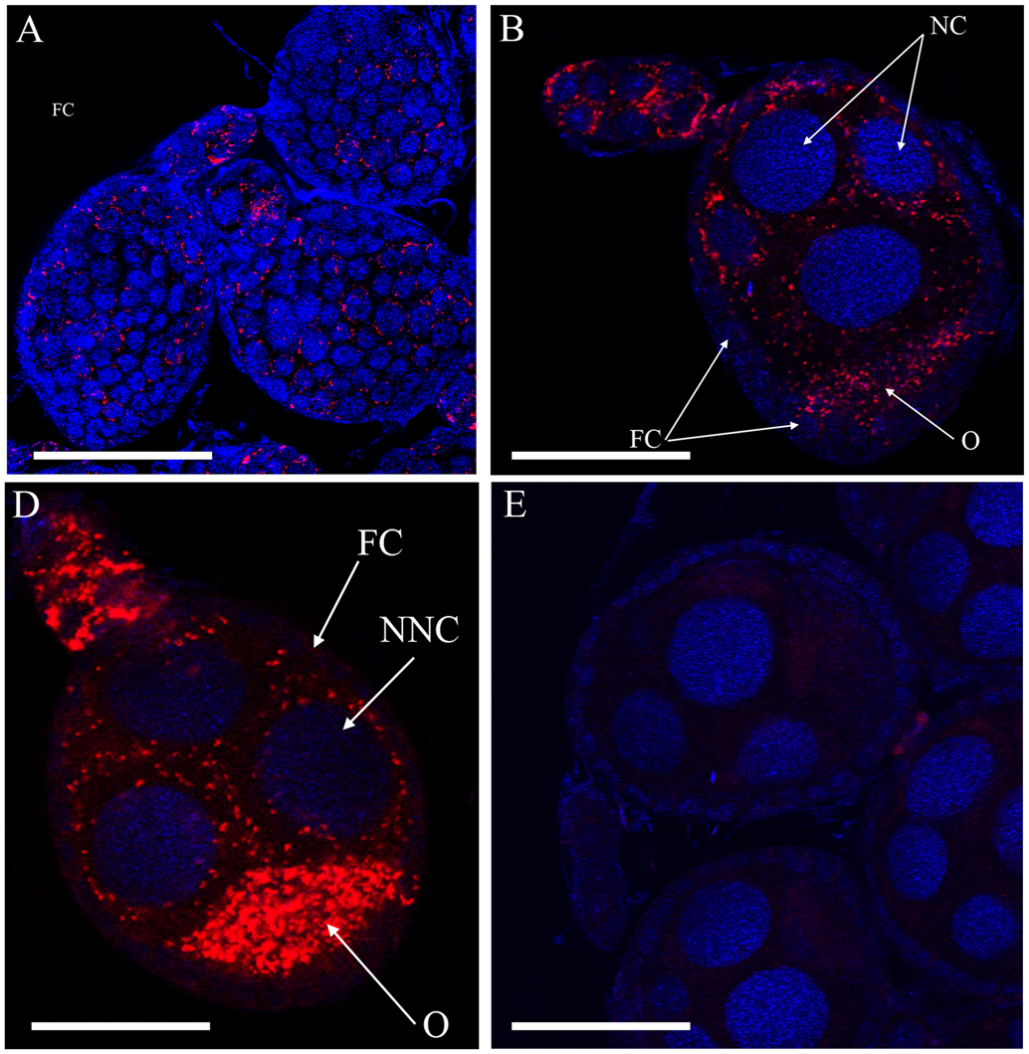
Confocal Microscopy of *Aedes albopictus* egg chambers infected with *Wolbachia*. (A) *Wolbachia* detected in follicular cells (FC). (B, C) Detection of *Wolbachia* in germ cells (NC, nurse cell; O, oocyte; NNC, nuclei of nurse cells). (D) View of egg chambers hybridized in the absence of *Wolbachia* probes. Nuclei (blue) are stained with DAPI, and *Wolbachia* (red) are stained by specific *rrs* gene FISH probes. Bar, 100 µm.

To monitor the bacteria in female reproductive tissues, the ovaries were dissected before vitellogenesis. Confocal images of the germarium and egg chambers revealed *Wolbachia* in all types of ovarian cells, including follicular and nurse cells, as well as in the future oocytes (Fig. 3). The highest density of bacteria was found in the future oocyte confirming a common feature of *Wolbachia*, which is to transfer from nurse cells into the oocyte through cytoplasmic dumping as has been shown in *Drosophila* [49]. As expected from the PCR results, no significant signal for *Acinetobacter* was found in the ovaries (not shown).

## Discussion

In recent years attempts have been made to investigate the possible use of native or genetically modified microbes to control pest arthropods and vector-borne diseases. The outcomes have varied considerably [8]. Greater knowledge about the behavior, persistence, and tissue tropism of microbes associated with vectors is essential to enhance the efficiency of paratransgenesis. Here, we investigated *Wolbachia* and the dominant cultivable bacteria in a colony of *Ae. albopictus* caught in La Réunion island during the 2005–2006 chikungunya epidemics. We found that the *Ae. albopictus* colony was infected by *Wolbachia* strains *w*AlbA and *w*AlbB. This is in accordance with what had been reported in studies of field-caught *Ae. albopictus*, where the prevalence of double infection by *Wolbachia* is commonly over 96% [43], [44]. We also report here for the first time the presence of cultivable bacteria of the genera *Acinetobacter*, *Comamonas*, *Delftia* and *Pseudomonas* in the whole bodies of *Ae. albopictus* individuals. In addition, GGE analysis yielded sequences closely related to the *Mesorhizobium* and *Stenotrophomonas genera*, as well as an uncultivable bacterium.

Genus-specific diagnostic PCR demonstrated the co-occurrence of the four cultivable genera (*Acinetobacter*, *Comamonas*, *Delftia* and *Pseudomonas*) together with *Wolbachia*, throughout four generations in both males and females, indicating persistent infections. The cultivable bacteria found here are widespread in nature, and can be found in water, soil and living organisms, including *Drosophila* [50]. Among the few data reported for mosquitoes, members of the *Pseudomonas* genus have been recorded in *Anopheles* [34], 36, *Aedes triseriatus* and *Culex pipiens* [37]. The genus *Acinetobacter* has been detected in the midgut of wild *Culex quinquefasciatus* [33]. When dissected tissues of mosquito females were subjected to diagnostic PCR detection, signals were found in the gut of all the cultivable bacteria. *In situ* hybridization with the specific oligonucleotide probes available made it possible to detect *Acinetobacter* in the lumen of the foregut and the anterior midgut of *Ae. albopictus* females. Interestingly, the PCR signal of *Acinetobacter* was also detected in the salivary glands, indicating the ability of this bacterium to spread throughout the insect body. This is consistent with the reported presence of *Acinetobacter* in the hemolymph of the glassy-winged Sharpshooter or *Homalodisca vitripennis* [51]. No cultivable bacteria were detected in the oocytes of the *Ae. albopictus* colony, ruling out the possibility of transovarial transmission. Canonical transovarial transmission of cultivable bacteria is in fact not a common event. In the mosquito, only the cultivable bacterium *Asaia* has been reported to be transmitted via the eggs of *Anopheles stephensi*, under the laboratory conditions [52]. Apart from transovarial transmission, other possible mechanisms of symbiont diffusion include proctophagy, the deposition of capsule containing microbes, and environmental acquisition [9], [53], [54], [55]. The presence of *Acinetobacter* in the gut and salivary glands, two organs where viruses are known to replicate, implies that the virus and the bacterium may share the same space or co-localize. Interestingly, a recent study has shown that *Acinetobacter* sp. strain KNF2022 was able to produce an antiviral compound with inhibitory effects on the tobacco mosaic virus [56]. The role that the cultivable bacteria found here may play in the bio-ecology or vectorial competence of *Ae. albopictus* needs to be investigated.

Like the cultivable bacteria, *Wolbachia* was found to be associated with both female and male *Ae. albopictus*. *Wolbachia* are obligate intracellular symbionts, and are generally passed transovarially from the female to her offspring during the early stages of oogenesis or embryogenesis. Consequently, reproductive tissues have been reported to be the main targets of *Wolbachia* infection in both arthropods and nematodes [57], [58], [59]. In *Ae. albopictus*, the development of diagnostic PCR revealed two *Wolbachia* strains, named *w*AlbA and *w*AlbB, that occur either separately or concomitantly in natural Asian populations [43], [60]. These two *Wolbachia* strains are transovarially transmitted, and induce cytoplasmic incompatibility (CI) in both native *Ae. albopictus* [45], [46], [47], [48], [61] and trans-infected *Ae. aegypti*, which is naturally devoid of *Wolbachia* [62]. Theoretical modeling has predicted that CI-inducing *Wolbachia* could be used to control the spread of mosquitoes [63], [64], this was achieved by empirical research in the medfly [17]. Here we show that the *Ae. albopictus* colony from La Réunion also harbored *Wolbachia* strains *w*AlbA and *w*AlbB, which are clearly transmitted during oogenesis, as high levels of specific *in situ* hybridization signals were found in ovarian cells. Indeed, *Wolbachia* was present in the cytoplasm of germ cells, and in that of all the cells in egg chambers, notably follicular cells, nurse cells and future oocytes. The high density of *Wolbachia* in the ovaries also supports these assumptions.

It has been established that *Wolbachia* can also infect somatic tissues [65], [66]. Dobson and co-workers [67] detected the WSP protein of *Wolbachia* in ovaries and testes, but also in heads, thoracic muscles, midguts, and Malpighian tubules. Genes encoding this protein could be present in *Wolbachia per se*, or could be part of a DNA fragment inserted into the host genome [68], [69], [70]. Recently, electron microscopic images of *Wolbachia* were reportedly detected in the salivary glands of the mosquito *Armigeres subalbatus* [71], [72]. Using specific oligonucleotide probes, we report here for the first time the detection of *Wolbachia* in the cell cytoplasm of the three lobes from salivary glands of *Ae. albopictus* females. This is a finding of major importance, as the salivary glands are crucial in virus transmission. The epidemiological consequences of this possible co-infection and potential cellular co-localization calls for careful investigation by arbovirologists, as it was shown recently that a strain of *Wolbachia* was able to reduce the lethal effect of viral pathogens of flies [14], indicating that *Wolbachia* has direct or indirect effects on the virus. Although the molecular mechanisms involved in this antiviral protection are still unknown, immunomodulation [73], [74], [75] and the induction of a reactive oxygen species burst [76] by *Wolbachia* infection could account for these effects on infectious agents.

In this study, we identified a persistent infection of obligate intracellular *Wolbachia* and cultivable bacteria, such as *Acinetobacter*, in *Ae. Albopictus*, a major vector of arboviruses. The bacteria effectively colonize the ovaries, gut, and salivary glands, organs that are essential for the replication and transmission of pathogens, such as arboviruses. Studies of the impact of these multiple infections on the vectorial competence of the mosquito are in progress.

## Materials and Methods

### Mosquitoes

Laboratory-reared *Ae. albopictus* was obtained from the DRASS (Direction Régionale des Affaires Sanitaires et Sociales) in La Réunion. *Aedes albopictus* Providence was collected in 2006, and the F2 to F5 generations were used in these experiments. Colonies were maintained at 28±1°C with a light:dark cycle of 16 h:8 h, and 80% relative humidity. Larvae were reared in pans containing 1 yeast tablet in 1 liter of tap water. Adults were provided with 10% sucrose solution *ad libitum*.

### Bacterial isolation

Adult mosquitoes were anaesthetized at 4°C, rinsed 3 times in sterilized water, surface disinfected by dipping in 70% ethanol for 5 min, and then rinsed five times in sterilized water, and once in sterilized NaCl 0.8%. Three whole mosquitoes were crushed in 250 µl sterilized NaCl 0.8%, two-fold diluted, and 100 µl of the resulting mix was plated on modified Luria Bertani agar medium (MLB: Bacto-trypthone 10 g.l^−1^, yeast extract 5 g.l^−1^, NaCl 5 g.l^−1^) and PYC medium (Peptone 5 g.l^−1^, yeast extract 3 g.l^−1^, CaCl_2_.2H_2_O 6 mM, pH 7.0). After incubating at 26°C, single colonies were streaked in the corresponding medium to check their purity. Purified isolates were cultured in liquid MLB at 26°C, stirred, and then stored in 25% glycerol at −80°C, until used.

### DNA extraction

To recover the various organs, adult females were dissected in PBS under a binocular microscope using needles. Five whole individuals or pools of 10 dissected organs were surface disinfected, as described above. Each whole insect sample was crushed in 200 µl (or 100 µl for the organ samples) of DNA extraction buffer (2% Hexadecyltrimethyl Ammonium Bromide, 1.4 M NaCl, 0.02 M EDTA, 0.1 M Tris pH 8, 0.2% 2-β mercaptoethanol) pre-warmed to 60°C. Homogenates were incubated for 15 min at 60°C. Proteins were removed in one volume of chloroform/isoamyl alcohol (24/1). DNA was precipitated at room temperature for 10 min with one volume of isopropyl alcohol. DNA pellet was washed once with 70% ethanol, air dried, and then dissolved in 30 µl of sterilized water. To extract the genomic DNA from the mosquito eggs, 30 to 70 mg of eggs were transferred into Eppendorf tubes, washed three times with sterilized water, surface disinfected in 70% ethanol for 5 min or dechorionized in 2.6% hypochlorite, before being rinsed twice in sterilized water. DNA extraction was then carried out as described above. For bacterial genomic extraction, an overnight culture was centrifuged at 12,000 x g, and the pelleted bacterial cells were handled using the DNeasy Tissue kit and QIAprep spin miniprep kit following the Manufacturer's instructions (QIAGEN, Courtaboeuf, France). For Plasmid DNA extraction, the QIAprep spin miniprep kit was used following the Manufacturer's instructions (QIAGEN, Courtaboeuf, France). All DNA samples were stored at −20°C until use.

### Diagnostic and quantitative PCR

The oligonucleotide primers used were synthesized by Invitrogen, and are listed in Table 1. PCR amplification of *rrs* genes using mosquito genomic DNA (60 ng) was performed in 25 µl of the reaction mixture in 1X polymerase reaction buffer (Roche), 200 µM of each deoxynucleoside triphosphate, 500 nM of each primer, 0.025 mg.ml^−1^ of T4 gene protein 32 (Roche), and 0.25 U of Expand DNA polymerase (Roche, France). *Wolbachia* was detected using specific primers targeting the 16S rDNA and *wsp* loci (Table 1) under the following conditions: 25 µl of the reaction mixture containing 60 ng of DNA template in 1X polymerase reaction buffer (Invitrogen), 1.5 mM MgCl_2_, 0.2 µM of each deoxynucleoside triphosphate, and 0.5 U of *Taq* polymerase (Invitrogen). Diagnostic PCR reactions were performed in a T gradient thermocycler (Biometra, France). Real-time quantitative PCR was performed using the LightCycler LC480 apparatus (Roche). The 20 µl reaction mixture contained 1X LightCycler DNA master SYBR green I (Roche), 300 nM of each primer, and 10 ng of template DNA. Amplifications consisted of 10 minutes at 95°C, followed by 40 cycles of 15 s at 95°C, 1 min at 60°C or 63°C for the *wsp* and *rrs* amplifications respectively, and a final elongation at 72°C for 30 s. Standard curves were drawn on DNA plasmids pQuantAlb [77] and TOPO 2.1-Acin, a TOPO 2.1 vector in which we have cloned a 280 bp-*rrs* gene fragment from *Acinetobacter calcoaceticus* (Table 1).

### DGGE

Ingeny PhorU (Apollo Instruments, Compiègne, France) system was used for DGGE analysis of the V3 PCR products as published [78]. Briefly, the 6% acrylamide gel contained a linear chemical gradient of urea and formamide from 35% to 65% (100% = 7 M urea and 40% [v/v] deionized formamide). PCR products (5 µg per well) were run in TAE buffer (40 mM Tris [pH 8.0], 20 mM acetic acid, 1 mM EDTA) at 60°C for 17 h at 100 V. After electrophoresis, the gels were immersed in SYBR green for 30 min at 4°C, rinsed in sterilized water, and then photographed under a UV lamp. Bands were excised, transferred to Eppendorf tubes, and washed three times with sterilized water. After all trace of liquid had been eliminated, 30 µl of water was added to the tubes, which were heated to 60°C for 30 min, and kept overnight at 4°C. Two µl of eluate were used for amplification. Products were purified (MinElute PCR purification kit, Invitrogen), and then direct sequenced using primers from the *rrs* V3 region (Genoscreen, Lille, France).

### Cloning, sequencing and accession numbers

PCR products were purified using QIAquick PCR purification kit (QIAGEN). ARDRA analysis was performed to screen 16S rDNA of bacterial isolates in 20 µl-reaction containing 200 ng DNA sample, 1X Buffer Tango™ and 10 U of each endonuclease R*saI* and H*haI* as recommended by the manufacturer (Fermentas, France). For cloning, selected products were inserted into the TOPO 2.1 vector, and used to transform the competent TOP10 *Escherichia coli* cells according to the procedure of the TOPO TA 2.1 cloning kit (Invitrogen). Clones containing DNA inserts were chosen for sequencing. Sequence analyses were performed using the Blastn program at the NCBI database (http://www.ncbi.nlm.nih.gov). Sequences have been deposited in the GenBank database (Table 2).

### Fluorescence *in-situ* hybridization (FISH)

Dissected organs (ovaries, salivary glands and guts) were fixed for 20 min in freshly prepared 4% formaldehyde in PBS, and then washed once with PBS. Cell line Aa23 infected with *Wolbachia* was also used, following the fixing procedure described in [78]. For *Acinetobacter*, an overnight culture of the isolate KZ-OAlM was centrifuged at 10,000 g, then 10^8^ pelleted cells were washed with PBS, and fixed as above. Hybridization was conducted using 200 ng probes in hybridization buffer [formamide 50%, SSC 5X, dextran sulfate 200 mg.l^−1^, poly(A) 250 µg.ml^−1^, salmon sperm DNA 250 µg.ml^−1^, tRNA 250 µg.ml^−1^, DTT 0.1 M, Denhartdt's solution 0.5X] at 37°C overnight. The probes were synthesized by Invitrogen and consisted of: two *Wolbachia* probes W2, 5′-CTTCTGTGAGTACCGTCATTATC-3′ [79] and Wol3, 5′-TCCTCTATCCTCTTTCAATC-3′ [80] 5′-end labeled with rhodamine; and two *Acinetobacter* probes ACA, 5′-ATCCTCTCCCATACTCTA-3′ [81] and Ac, 5′-GCGCCACTAAAGCCTCAAAGGCC-3′ [82] 5′-labelled with alexa488. Samples were washed twice in 1X SSC-10 mM DTT and twice in 0.5X SSC-10 mM DTT at 55°C for 15 min. Finally, samples were rinsed in PBS, mounted on a glass slide with glycerol alone or with 1 µg.ml^−1^ DAPI (4′, 6′-diamidino-2-phenylindole) and viewed under a fluorescent (AXIO Imager.Z1, Zeiss) and a confocal microscope (LSM510, Zeiss) at the Microscopy Centre of University Lyon I.

## Supporting Information

Figure S1 DGGE profiles of bacterial *rrs* V3 segments from *Aedes albopictus*. Females and males from generations F2 to F5 (whole insect body), dissected ovaries (OV), gut (G), and salivary glands (SG). wRi, *Wolbachia* strain purified from *Drosophila simulans* Riverside [89]. Numbers correspond to sequenced bands (Table 2).
(0.37 MB TIF)
