# Supplementary figures and images for: Persistent Wolbachia and Cultivable Bacteria Infection in the Reproductive and Somatic Tissues of the Mosquito Vector Aedes albopictus
Source: PLoS One. 2009 Jul 27;4(7):e6388. doi: 10.1371/journal.pone.0006388 (PMC2712238; doi:10.1371/journal.pone.0006388)

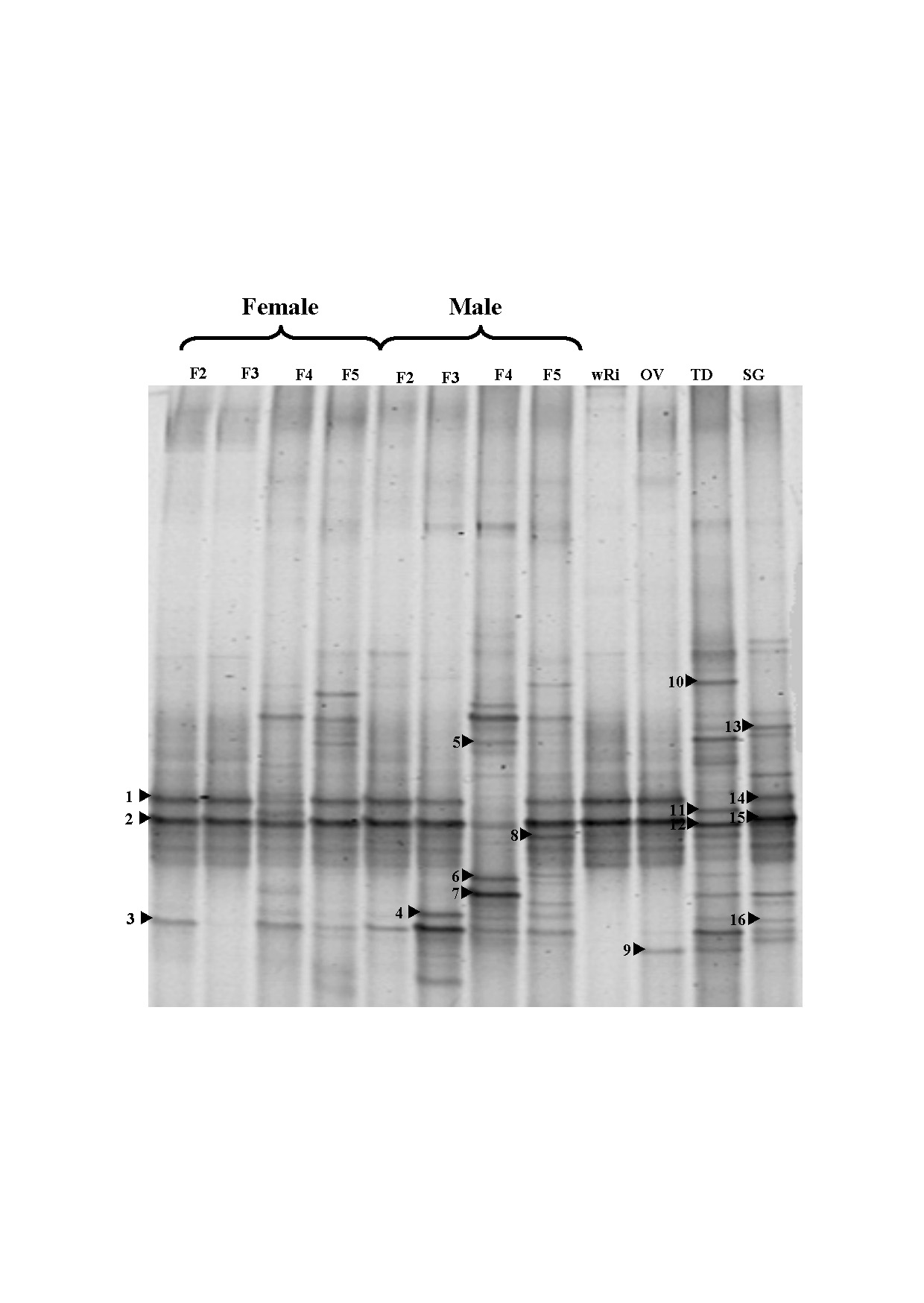

Supplement: Figure S1 — DGGE profiles of bacterial rrs V3 segments from Aedes albopictus. Females and males from generations F2 to F5 (whole insect body), dissected ovaries (OV), gut (G), and salivary glands (SG). wRi, Wolbachia strain purified from Drosophila simulans Riverside [89]. Numbers correspond to sequenced bands (Table 2). (0.37 MB TIF) [file pone.0006388.s001.tif]
